# Effect of Patency File on Transportation and Curve Straightening in Canal Preparation with ProTaper System

**DOI:** 10.1155/2013/704027

**Published:** 2013-09-12

**Authors:** Seyed Mohsen Hasheminia, Nastaran Farhadi, Ali Shokraneh

**Affiliations:** ^1^Torabinejad Dental Research Center and Department of Endodontics, School of Dentistry, Isfahan University of Medical Sciences, Isfahan 8174755153, Iran; ^2^Torabinejad Dental Research Center and Department of Oral and Maxillofacial Radiology, School of Dentistry, Isfahan University of Medical Sciences, Isfahan 8174673461, Iran

## Abstract

The aim of this *ex vivo* study was to evaluate the effect of using a patency file on apical transportation and curve straightening during canal instrumentation with the ProTaper rotary system. Seventy permanent mandibular first molars with mesiobuccal canals, measuring 18–23 mm in length and with a 25–40° curvature (according to the Schneider method), were selected. The working lengths were determined and the teeth were mounted and divided into two experimental groups: (A) prepared by the ProTaper system without using a patency file (*n* = 35) and (B) prepared by the ProTaper system using a patency file (*n* = 35). Radiographs taken before and after the preparation were imported into Photoshop software and the apical transportation, and curve straightening were measured. Data were analyzed using independent *t*-test. Partial correlation analysis was performed to evaluate the relationship between the initial curvature, transportation, and curve straightening (*α* = 0.05). Using a patency file during canal preparation significantly decreased both apical transportation and curve straightening (*P* < 0.001). There were significant relationships between the angle of curvature, transportation and curve straightening in pairs (*P* < 0.001). Apical patency is recommended during root canal preparation with the ProTaper rotary system.

## 1. Introduction

Cleaning and shaping the root canal system is an important step in the success of root canal therapy [1]. Three-dimensional maintenance of the original shape of the root canal is necessary during canal preparation. To achieve this goal, cleaning and shaping should be performed circumferentially, foramen transportation should not occur, and apical foramen should be maintained in its original position [1]. During the root canal instrumentation pulpal and dentinal debris can block the apical third of the root canal, which can increase the chance of ledge formation, transportation, and perforation [2]. These procedural errors can be prevented with the use of a patency file during instrumentation.

NiTi rotary instruments obviously decrease several clinical complications, such as canal blockage, ledge formation, transportation, and perforation; they also reduce operator fatigue and the time required for canal preparation [3–6]. ProTaper is one of the NiTi rotary systems, with progressive tapering and a convex triangular cross-sectional cutting blade designed for increased flexibility and cutting efficiency. This system has great applicability in curved canals.

Several studies have evaluated canal transportation and curve straightening of ProTaper files. In one study, the ProTaper system resulted in more canal transportation and curve straightening than the Flex Master system, but the difference was not statistically significant [7]. Another study demonstrated no differences between the ProTaper and GT systems in the extent of canal transportation [8]. In addition, H. H. Javaheri and G. H. Javaheri [9] reported that ProTaper files lead to more apical transportation and curve straightening than the Hero 642 and RaCe systems. Great efforts have been made to decrease the transportation caused by ProTaper files, including combining Pathfiles with ProTaper files [10].

Some studies have suggested the use of a patency file for most rotary instrumentations to remove accumulated debris and help maintain working length [2, 11]. However, there is still controversy about the use of a patency file. Moreover, one study demonstrated that the use of patency files did not prevent preparation errors [12]. Therefore, the aim of this study was to evaluate the effect of patency files on the apical transportation and curve straightening during instrumentation with the ProTaper rotary system.

## 2. Materials and Methods

Ninety permanent mandibular first molars with intact crowns and roots and completely developed apices were used in this experimental study. The teeth had mesiobuccal canals, measuring 18–23 mm in length with a 25–40° curvature according to the Schneider method [13] with a snug fit of no. 15 K-file. The teeth had been extracted for periodontal or prosthetic reasons. All the samples were cleaned by scaling and soaking in 2.5% sodium hypochlorite for 24 hours. To prevent the superimposition of roots, distal roots were resected. The samples were stored in saline at 4°C until use.

Anatomic access cavities were prepared with round diamond burs and an Endo-Z bur (Dentsply Maillefer, Ballaigues, Switzerland) in a high-speed handpiece; no. 08 K-files (Mani Inc., Utsunomiya, Japan) were inserted into the mesiobuccal canals until the tip of the files was seen just at the apical foramen. The file length was measured with a digital caliper to the nearest 0.001 mm (Mitutoyo, Tokyo, Japan); 0.5 mm short of this measurement was recorded as the working length. Only teeth with a length of 18–23 mm in length were included. The tip of the reference cusps was reduced until each canal had an 18 mm working length.

Each tooth was mounted on an acrylic block with a no. 15 K-file in the mesiobuccal canal so that the external walls of each block were parallel to the tooth long axis. Two 10 mm rectangular orthodontic wires were also mounted on the mesial and distal side of each tooth parallel to the tooth long axis. The blocks were placed on a Pentamix supporting post (3M ESPE, Seefeld, Germany) with the X-ray cone to allow exact parallelism. A Rinn-Endo-ray film holder (Dentsply/Rinn Corporation, Elgin, IL, USA) was used to keep the receptor of the digital radiograph perpendicular to the beam during all the exposures and to provide reproducible exposition geometry. The standard geometric configuration was fixed at 25 cm source-to-object distance, and radiographs were obtained for each sample at three angles of zero, 45, and 90 degrees mesially without any vertical angulations. The radiographic images of each sample were obtained with the Digora SPPs (Soredex Corporation, Helsinki, Finland), and each image was saved as a JPEG file for later analysis. The angles of curvatures of 240 radiographs were measured and recorded. Among the three images obtained from each sample, the one with the greatest curvature in the range of 25 to 40 degrees was selected as a reference image.

The seventy teeth were divided into two experimental groups randomly: (A) prepared by the ProTaper system without using a patency file (*n* = 35) and (B) prepared by the ProTaper system using a patency file (*n* = 35). Independent *t*-test showed no statistically significant differences in the degree of curvatures of the samples between the two groups (*P* = 0.396). The remaining twenty teeth were included in the negative control group for verification of accuracy of radiographs before and after preparation.

The mesiobuccal canals of the samples in groups A and B were prepared, according to the ProTaper manufacturer's instructions (Table 1), with low-torque motors with a torque control and a constant speed of 300 rpm (ATR Tecnika, Advanced Technology Research, Pistoia, Italy). Groups A and B differed in the performance of patency files. In group B, after using each file up to the working length, a no. 8 K-flex file (Kerr Sybron, Bretton, Peterborough, UK) was passed through the apical foreman one mm more than the working length for three times. In the negative control group, another radiograph was taken with no. 15 K-file in the mesiobuccal canal without any preparation.

After using each file during the canal preparation, each canal was irrigated with 2 mL of 2.5% sodium hypochlorite. After the completion of canal preparation, the root canal was passively irrigated with 10 mL of 2.5% sodium hypochlorite. After final irrigation, no. 30 K-files were inserted into the mesiobuccal canals and the radiographs were taken exactly like the initial radiographs.

The initial and secondary radiographs were exported to Adobe Photoshop software 3.0 (Adobe Systems Inc., San Jose, CA, USA). AutoCAD 2005 computer program (Autodesk Inc., San Rafael, CA, USA) was used to draw the central axis of both files. The two digital images were superimposed by using the two orthodontic wires. In the two experimental groups, the angle between the tips of no. 15 and no. 30 files (curve straightening) and the distance between the tips of these files (transportation) were measured by a radiologist (NF) who was blind to the preparation techniques used.

Data were obtained and first verified with the Kolmogorov-Smirnov test for the normality of data distribution and the Levene test for the homogeneity of variances. Independent *t*-test was performed to compare curve straightening and transportation between the two groups. In addition, partial correlation analysis was performed to evaluate the relationship between the initial curvature, transportation, and curve straightening of the canals in pairs by adjusting the groups. SPSS 10.0 Software (SPSS Inc., Chicago, IL, USA) was used for statistical analysis. Statistical significance was set at a confidence level of 95%.

## 3. Results

**Figure 1:**
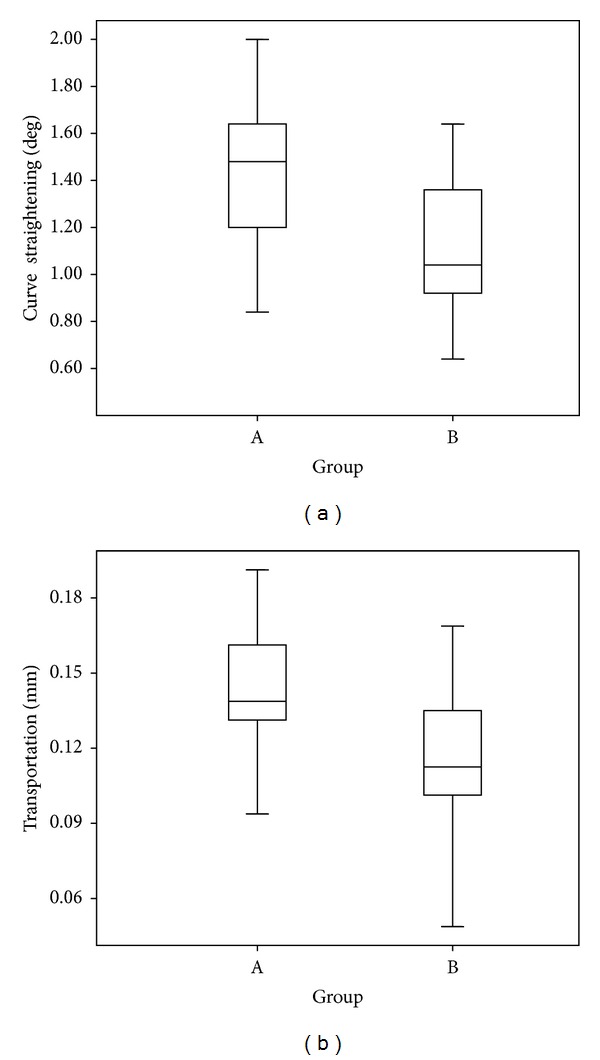
Box plots of transportation and curve straightening in the two groups. Group A: preparation without using a patency file and group B: preparation with the use of a patency file.

Independent *t*-test showed that using a patency file during canal preparation with the ProTaper system significantly decreased the transportation and curve straightening (*P* < 0.001). These results are summarized in Table 2 and Figure 1.

**Figure 2:**
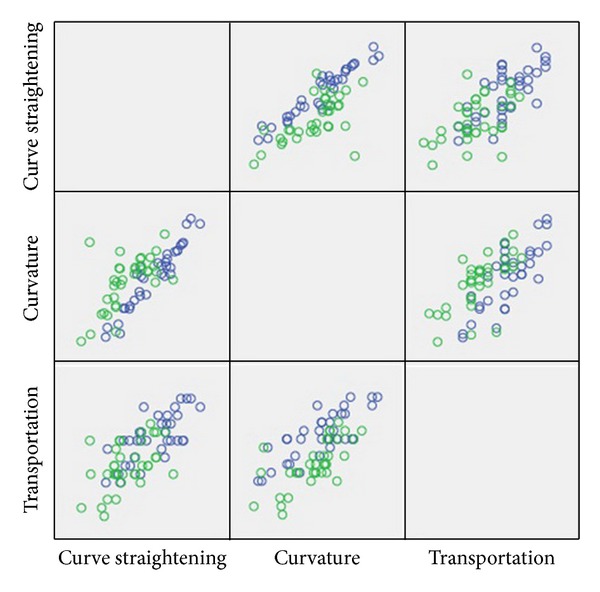
A scatter plot of initial curvature, curve straightening, and transportation. Significant correlations were detected. Group A (blue): preparation without using a patency file and group B (green): preparation with the use of a patency file.

Partial correlation analysis indicated a statistically significant relationship between the angle of curvature and transportation (correlation coefficient = 0.741, *P* < 0.001), the angle of curvature and curve straightening (correlation coefficient = 0.813, *P* < 0.001), and transportation and curve straightening (correlation coefficient = 0.620, *P* < 0.001). These correlations have been shown in Figure 2.

## 4. Discussion

A large array of techniques has been developed for evaluating the shaping ability of hand and rotary files. Using pictures from resin blocks simulating root canal before and after preparation is one of these techniques. Cross-sectioning at different levels is another technique performed on extracted teeth [14]. The main disadvantage of the latter technique is the inability to record original canal configuration before instrumentation [15]. To overcome this disadvantage, Bramante et al. [14] described a technique to record the original shape of the canal before preparation. However, this approach also leads to the loss of samples because of gaps between the sections of the root [16, 17]. Longitudinal cleavage of the teeth is another technique to evaluate the efficacy of instruments to remain centered during preparation. Backman et al. [18] used drawing of projected radiographic images of files to compare the position of master apical file with the position of initial file. Sydney et al. [19] described a radiographic platform for evaluation of canal transportation. In their method each film was exposed twice: before and after canal preparation with a file in the canal; then, it was developed and canal transportation was measured. Another technique for evaluating the shaping ability of different instrumentations is superimposition of two radiographs before and after preparation to measure canal transportation and curve straightening [20, 21]. However, its main limitation is the inability of conventional radiographs to show the maximum curvature of root canals [18, 20]. To overcome this limitation in the present study, three radiographs from three different horizontal angles (zero, 45, and 90 mesial degrees) were taken from each tooth in order to determine maximum curvature of the mesiobuccal canal. Then, the image which demonstrated the maximum curvature was used as a reference and the secondary radiograph was obtained from the same angle. The newest techniques for evaluation of shaping ability of rotary instrumentation are microtomography [22, 23], high-resolution computed tomography [10, 24], and cone-beam computed tomography [7], which are nondestructive and show more details [25]. These techniques are more expensive and need more equipments and large radiation doses.

Several studies have surveyed canal preparation with the ProTaper rotary instrument. These studies have yielded controversial results in relation to canal transportation due to preparation with the ProTaper system. In two studies, canal transportation and curve straightening of the ProTaper files were more than those of the Hero 642 system [9, 26], unlike another study which equal amounts for both [7]. In addition, two other studies have shown that transportation of the ProTaper files was more than that with the RaCe files [9, 27]. However, Guelzow et al. [26] demonstrated that the extent of transportation with the ProTaper system is similar to that with the RaCe system. In summary, with respect to different studies, it seems that the ProTaper system is a rotary system, which results in the greatest amount of canal transportation. In order to reduce the extent of this transportation, canal preparation with the ProTaper rotary system was combined with the Pathfiles [10]. Although transportation of this combination was less than that with hand instrumentation, it was more than that with the Twisted file system [10].

Another recommendation for reducing the degree of transportation was the use of patency files although it is still controversial. In the present study, no. 8 K-flex file was used as a patency file based on the findings of Gutiérrez et al. [28], Gonzalez Sanchez et al. [29], and Goldberg and Massone [12]. Gutiérrez et al. [28] reported that cementum fractures and dentinal chips occur at the apex after the penetration of a no. 15 file through the main foramen. Gonzalez Sanchez et al. [29] found no transportation in the majority of samples when no. 08 K-Flex files and no. 10 stainless steel reamers were used. Goldberg and Massone [12] assessed the effect of patency files on canal transportation. They reported that canal patency has no effect on canal transportation and transportation is initiated even after the use of no. 10 file as a patency file.

In the present study, canal transportation decreased as a result of using a patency file, consistent with the results reported by Hasheminia and Shafiee Ardestani [30]. They demonstrated that the use of patency files in the preparation of root canals with the use of the passive step-back technique decreases the degree of transportation. In contrast, Tsesis et al. [31] evaluated the transportation degree in the preparation of root canals with low-speed rotary instruments and balanced-force technique with the use of hand instruments with and without the use of a patency file. They reported that patency files had no effect on the transportation of root canals one, two, and four mm short of the working length. Because of these inconsistent results, more investigations are recommended on the subject with the use of ProTaper and other rotary instruments and new techniques such as microtomography, high-resolution computed tomography, and cone-beam computed tomography.

The results of the present study showed good correlation between the angle of canal curvature and transportation, consistent with the results reported by Dummer's group [32–40]. They showed the effect of canal geometry on the outcome: the more severe the angle of the curve, the more severe the canal transportation. Their studies were performed on resin blocks. Contrary to previous findings, another study demonstrated that there was no relationship between the amount of transportation and the angulation of root canals [41]. The authors of the latter study suggested that this might be attributed to the low angulations of the root canals or to the small size of the sample.

## 5. Conclusion

Apical patency is recommended during the root canal preparation by ProTaper rotary system because patency files reduce apical transportation and curve straightening.

## Figures and Tables

**Table 1:** Sequences of using ProTaper rotary instruments according to manufacturer's recommendations.

File type	Length
S_1_	1/3 to 2/3 coronal
SX	2/3 coronal
S_1_, S_2_, F_1_, F_2_, F_3_	Working length

**Table 2:** Means ± standard deviations of transportation and curve straightening of each group.

	Number	Transportation (millimeter)	Curve straightening (degree)
Group A	35	0.14 ± 0.03	1.41 ± 0.30
Group B	35	0.11 ± 0.03	1.10 ± 0.30
Control group	20	0.00 ± 0.00	0.00 ± 0.00

Group A: preparation without using a patency file and group B: preparation with the use of a patency file.
